# Vascular ATGL-dependent lipolysis and the activation of cPLA_2_–PGI_2_ pathway protect against postprandial endothelial dysfunction

**DOI:** 10.1007/s00018-024-05167-6

**Published:** 2024-03-12

**Authors:** M. Sternak, M. Stojak, T. Banasik, A. Kij, A. Bar, M. Z. Pacia, K. Wojnar-Lason, N. Chorazy, T. Mohaissen, B. Marczyk, I. Czyzynska-Cichon, Z. Berkimbayeva, A. Mika, S. Chlopicki

**Affiliations:** 1 Jagiellonian Centre for Experimental Therapeutics (JCET), Jagiellonian University, Bobrzynskiego 14, Krakow, Poland; 2 Medical College, Chair of Pharmacology, Jagiellonian University, Grzegorzecka 16, Krakow, Poland; 3 Doctoral School of Exact and Natural Sciences, Jagiellonian University, Lojasiewicza 11, Krakow, Poland; 4 Department of Environmental Analysis, Faculty of Chemistry, University of Gdansk, Wita Stwosza 63, Gdansk, Poland; 5 Department of Pharmaceutical Biochemistry, Medical University of Gdansk, Debinki 1, Gdansk, Poland

**Keywords:** Lipid droplets, Lipolysis, Endothelium, Endothelial-induced vasodilation, Atglistatin, ATGL

## Abstract

Adipose triglyceride lipase (ATGL) is involved in lipolysis and displays a detrimental pathophysiological role in cardio-metabolic diseases. However, the organo-protective effects of ATGL-induced lipolysis were also suggested. The aim of this work was to characterize the function of lipid droplets (LDs) and ATGL-induced lipolysis in the regulation of endothelial function. ATGL-dependent LDs hydrolysis and cytosolic phospholipase A_2_ (cPLA_2_)-derived eicosanoids production were studied in the aorta, endothelial and smooth muscle cells exposed to exogenous oleic acid (OA) or arachidonic acid (AA). Functional effects of ATGL-dependent lipolysis and subsequent activation of cPLA_2_/PGI_2_ pathway were also studied in vivo in relation to postprandial endothelial dysfunction.

The formation of LDs was invariably associated with elevated production of endogenous AA-derived prostacyclin (PGI_2_). In the presence of the inhibitor of ATGL or the inhibitor of cytosolic phospholipase A_2_, the production of eicosanoids was reduced, with a concomitant increase in the number of LDs. OA administration impaired endothelial barrier integrity in vitro that was further impaired if OA was given together with ATGL inhibitor. Importantly, in vivo, olive oil induced postprandial endothelial dysfunction that was significantly deteriorated by ATGL inhibition, cPLA_2_ inhibition or by prostacyclin (IP) receptor blockade.

In summary, vascular LDs formation induced by exogenous AA or OA was associated with ATGL- and cPLA_2_-dependent PGI_2_ production from endogenous AA. The inhibition of ATGL resulted in an impairment of endothelial barrier function in vitro*.* The inhibition of ATGL-cPLA_2_-PGI_2_ dependent pathway resulted in the deterioration of endothelial function upon exposure to olive oil in vivo. In conclusion, vascular ATGL-cPLA_2_-PGI_2_ dependent pathway activated by lipid overload and linked to LDs formation in endothelium and smooth muscle cells has a vasoprotective role by counterbalancing detrimental effects of lipid overload on endothelial function.

**Supplementary Information:**

The online version contains supplementary material available at 10.1007/s00018-024-05167-6.

## Introduction

Adipose triglyceride lipase (ATGL) has been identified as a key enzyme of mammalian lipolysis, involved in the hydrolytic cleavage of triglycerides (TG) into free fatty acids (FFA) and diacylglycerols. ATGL mainly localizes to TG-rich intracellular lipid droplets and it is predominantly expressed in adipose tissue. However, ATGL was also found to a lesser extent in a variety of other tissues and organs, including kidney, liver, skeletal muscle, immune cells lung and heart [1–7]. Studies with the use of genetic mouse models with tissue-specific overexpression or deletion of ATGL as well as with the use of the recently developed ATGL-specific inhibitor (atglistatin) gave unprecedented insight into the pathophysiological role of ATGL.

Quite intriguingly, ATGL-dependent lipolysis was reported to display detrimental or beneficial role, depending on targeted organ or tissue. In the liver, ATGL-mediated lipolysis plays an important function in hepatic lipid homeostasis and ATGL inhibitor, atglistatin substantially reduced high-fat diet-induced hepatosteatosis, obesity, liver inflammation and hepatic fibrosis [1]. Moreover, atglistatin effectively diminished the metabolic consequences of obesity such as insulin resistance and non-alcoholic fatty liver disease (NAFLD) in mice fed a high-fat diet [2].

In relation to cardiac pathology, number of reports demonstrated the beneficial effects of ATGL inhibition. Botterman et al. demonstrated that inhibiting lipolysis with atglistatin was able to improve cardiac function after myocardial infarction [4]. Inhibition of ATGL afforded also the cardioprotective effect on catecholamine-induced cardiac damage [5] and protected against heart failure induced by pressure overload [6, 7].

In other organs, targeting ATGL had also therapeutic or detrimental effects. For example, in severe burn injuries resulting in hypermetabolic response together with hyperlipidemia and fatty liver development, atglistatin protected against the development of fatty liver and post-burn injury[3]. On the other hand, ATGL-mediated lipid hydrolysis had an important role in bronchial regeneration. Deletion of the gene encoding ATGL induced substantial triglyceride accumulation, decreased mitochondrial numbers and decreased mitochondrial respiration in club cells [8]. Finally, loss of the *Atgl* gene induced the development of spontaneous pulmonary neoplasia, which progressed to adenocarcinoma [9].

Despite the progress in the studies related to the role of ATGL in various organs and diseases, still little is known about the role of ATGL-dependent lipolysis in vascular wall linked to LDs formation. On the other hand, it is not clear whether the vascular ATGL pathway has a beneficial or detrimental role in the maintenance of endothelial function.

Initially, LDs formation in the vascular wall was related to the pathophysiology of atherosclerosis [10, 11]. Recently, however, in an elegant study by Kuo et al. it was demonstrated that LDs metabolism protected endothelial cells from lipotoxicity and provided fatty acids for mitochondrial function and transport to adjacent cells [12]. In a number of recent reports from our group, the biochemical content of LDs in endothelial cells was analyzed using Raman spectroscopy [13, 14] and it was demonstrated that endothelial inflammation in cultured endothelial cells or in the isolated murine aorta was invariably associated with the formation of LDs suggesting that their formation is an integral component of vascular inflammation [15, 16].

Noteworthy, the role of LDs in leukocytes in the generation of eicosanoids is well accepted [17, 18] as well as their modulatory role in inflammatory and immune responses of leukocytes [19]. In fact, it is well known that inflammatory response in leukocytes can be amplified not only by the enzymatic decomposition of membrane phospholipids but also by the hydrolysis of arachidonic acid (AA)-rich triglycerides stored in LDs in some cells.

Vascular wall homeostasis and inflammation are highly regulated by eicosanoids [17, 19–23]. Although it is already known that LDs formation may be linked to PGI_2_ generation in response to a wide range of stimuli [24] yet, the functional consequences of LDs formation and eicosanoids generation linked to LDs formation in terms of endothelial function have not been elucidated.

The aim of this work was to investigate the functional role of LDs in the regulated formation of AA-derived eicosanoids via ATGL-cPLA_2_ pathway in the vascular wall. Firstly, we characterized in detail ATGL-dependent lipid droplets formation and hydrolysis, cytosolic phospholipase A_2_-dependent eicosanoids production in aorta, endothelial and smooth muscle cells exposed to exogenous oleic acid (OA) and AA, using fluorescence and Raman imaging as well as LCMS/MS-based eicosanoids analysis. Then, we characterized the functional role of ATGL-dependent lipolysis and subsequent activation of cPLA_2_–PGI_2_ pathway offsetting postprandial endothelial dysfunction in vivo.

## Materials and methods

### Animals and aorta isolation

C57BL/6 mice (wild type) at the age of 8–12 weeks were kept under controlled conditions (22–24 °C, 55% humidity, 12 h day/night rhythm with free access to food and water until the day of the experiment. All experimental procedures involving animals were conducted according to the Guidelines for Animal Care and Treatment of the European Communities and the Guide for the Care and Use of Laboratory Animals published by the US National Institutes of Health (NIH Publication No. 85-23, revised 1996). All procedures were approved by the second Local Ethical Committee on Animal Experiments in Krakow. Mice were euthanized by an intraperitoneal injection of a mixture consisting of ketamine and xylazine (100 mg ketamine+10 mg xylazine/kg). The chest was opened and the thoracic aorta was removed and transferred into Krebs–Henseleit buffer. Subsequently, the aorta was cleaned from surrounding adipose tissue and transferred into medium (minimal essential medium, MEM, Sigma Aldrich) with the addition of 1% vitamins (Sigma Aldrich), 1% antibiotics (penicillin 10,000 U/mL and streptomycin 10,000 μg/mL, ThermoFisher Scientific) and 1% non-essential amino acids (Sigma Aldrich). The aorta was incubated with oleic acid (OA, Cayman Chemical) at a concentration of 500 μM or 1 mM in the presence or absence of lipolysis inhibitor, atglistatin (50 µM, Cayman Chemical) and phospholipase A_2_ inhibitor, arachidonyl trifuoromethyl ketone (AACOCF3, 10 µM, Abcam) for 4 h or 24 h. Aortic samples were incubated at 37 °C and 5% CO_2_. For immunofluorescence staining and Raman imaging of the aorta en face, the resected and split-open arteries were tightly glued to the Cell-Tak® (Corning)-coated microscopic glasses and calcium fluoride surface, respectively. Subsequently, the tissues were preserved by a 15-min soak in 4% paraformaldehyde (fluorescence imaging) or a 10-min soak in 4% buffered formalin (Raman imaging).

### Cell culture

Human aorta endothelial cells line (HAEC) was obtained from Lonza (Basel, Switzerland). Vascular smooth muscle cell line (MOVAS) was purchased from American Type Culture Collection (ATCC, Rockville, Maryland, MD, USA). HAECs were maintained in supplemented endothelial growth medium EGM-2 (Lonza, Basel, Switzerland), MOVAS cells were cultured in DMEM supplemented 10% (v/v) FBS (both from Gibco, Scotland, UK). Cultured were maintained at 37 °C in a humidified atmosphere of 5% CO_2_—95% air. When cells achieved 90% confluence the medium was changed and cells were treated with 100 μM deuterated oleic acid (OAd34, Sigma Aldrich) or deuterated arachidonic acid (AAd8, 25 µM, Cayman Chemical) in the presence or absence of lipolysis inhibitor, atglistatin (50 µM), phospholipase A_2_ inhibitor (AACOCF3, 10 µM, Abcam), COX-1 inhibitor (SC-560, Cayman Chemical) or COX-2 inhibitor (Dup-697, Cayman Chemical) for 4 h or 24 h. Oleic acid (Cayman Chemical) for the experiments was freshly saponified using 100 mM NaOH and BSA-conjugated (10% in DPBS, low endotoxin, fatty acid-free, suitable for cell culture, sterile-filtered; Sigma Aldrich). After 4 h or 24 h of incubation cells were fixed for 4 min with 4% paraformaldehyde. Fixed cells were washed 3 times with DPBS and stored in DPBS at 4 °C until the execution of the measurements.

### MTS assay

The effect of arachidonic acid and oleic acid on HAEC and MOVAS cell viability was determined by the MTS tetrazolium substrate (CellTiter 96 AQueous Non-Radioactive Cell Proliferation Assay, Promega, Madison, WI, USA) according to the manufacturer's protocol. The absorbance was measured at 490 nm using a spectrophotometer (Synergy 4, BioTek, Winooski, VT, USA). Experiments were performed 3 times in eight technical replicates. Cellular metabolic activity changes were assessed after the HAEC and MOVAS cells were incubated for 24 h with the tested compound.

### Immunostaining of the aorta en face

#### LDs and CD31 detection

Thoracic aorta isolated from 8-12 weeks old C57BL/6 mice was dissected and cut longitudinally. Aortic samples were pinned down with (endothelial cells (EC) facing upward, washed with DPBS 3 times and fixed with 4% paraformaldehyde for 15 min. Fixed samples were further blocked with TNB blocking buffer (0.1 M Tris–HCl pH 7.5, 0.15 M NaCl, and 0.5% (w/v) blocking reagent (PerkinElmer) for 3–4 h, and then incubated with CD31 antibody (Abcam, 1:50) diluted in TNB blocking buffer overnight at 4 °C. As a secondary antibody, Alexa Fluor 647 goat-anti-rabbit (Jackson Immuno Research; 1:200) was used at room temperature for 3 h. BODIPY 493/503 (Invitrogen) diluted in DPBS at the final concentration of 0.1 mg/mL was applied for 1 h to delineate LDs, and Hoechst 33342 (Sigma Aldrich; 1:1000) was used to highlight nuclei. Samples were visualized on a CQ1 Confocal Quantitative Image Cytometer (Yokogawa, Japan) at 40 × magnification.

### Raman imaging of aorta en face

Raman imaging was carried out using a WITec Confocal Raman Imaging system (WITec alpha300, Ulm, Germany) supplied with a UHTS 300 spectrograph (600 grooves mm^−1^ grating, resolution of 3 cm^−1^) and a CCD detector (Andor, DU401A-BV-352). The air-cooled solid-state laser with an excitation wavelength of 532 nm was coupled to the microscope via an optical fiber with a diameter of 50 μm. Raman spectra of tissues were collected with the application of a 63 × water immersive objective (Zeiss Fluor, Numerical Aperture, NA = 1.0), using maximum laser power at the sample position (ca. 30 mW) and 0.4 s exposure time per spectrum. The nominal minimal lateral and depth resolution for our setup is 0.32 and 0.53 μm, respectively and sampling density of 0.38–0.50 and 0.5–1.0 μm in *x*/*y* and *z* direction, respectively, was used. Depth profiling of the tissue was obtained by multiple imaging of the same line in several layers of the sample. The distribution images collected at different depths present the relative intensity of a studied component in the tissue. Data matrices were analysed using WITec Project software (background subtraction using a polynomial of degree 2 and the automatic removal of cosmic rays). The analysis of the spectra was supported by a Cluster Analysis (CA) (K-means, Manhattan distance, WITec Project Plus). This approach enabled data grouping into classes and extraction of average spectra reflecting the major compartments inside tissues. For the study of heterogeneity of LDs observed in in situ endothelial cells within isolated blood vessels treated with OA or OAd34, the single Raman spectra were extracted from the centre of each LD and then averaged. The OPUS 7.2 program was used for calculations of the integral intensity of the bands at ca. 2940 (reflecting the overall content of lipids), 1745 (esters), 1660 (level of unsaturation), 1445 (level of unsaturation), 702 (cholesterols) cm^−1^ in the 2815–3033, 1733–1767, 1563–1712, 1394–1505 and 695–709 cm^−1^ spectral ranges, respectively. Integration was performed using method D, OPUS 7.2: the integral was defined by the wavenumber limits and the horizontal baseline was determined by a chosen baseline point.

### LC–MS/MS eicosanoid analysis

Selected eicosanoids were quantified in a medium collected after aorta incubation and cell culturing using an LC–MS/MS-based method with the application of an already published methodology [25]. In short, each sample was spiked with a mixture of internal standards and gently mixed. Next, samples were cleaned up using liquid–liquid extraction by the addition of acidified ethyl acetate. After vigorous shaking and centrifugation, the organic layer was transferred to a fresh tube and evaporated to dryness under a nitrogen stream (37 °C). The dry residue was dissolved in ethanol and after centrifugation, clean samples were injected into an LC–MS/MS system comprising a Nexera UFLC (Shimadzu, Kyoto, Japan) ultrapressure liquid chromatograph combined with a QTrap 5500 (Sciex, Framingham, Massachusetts, USA) triple quadrupole mass spectrometer. The detailed conditions of chromatographic separation and mass spectrometric detection as well as the sample preparation procedure were described in our previous work [25].

The lipid extract from HAEC was subjected to AA and AAd8 LC–MS/MS analysis using an Ultimate 3000 UHPLC (Dionex, Sunnyvale, California, USA) liquid chromatograph and TSQ Quantum Ultra triple quadrupole mass spectrometer (Thermo Fisher Scientific). The best chromatographic separation was achieved on an Acquity UPLC BEH C18 (3.0 × 100 mm, 1.7 μm, Waters, Milford, Massachusetts, USA) analytical column. The mobile phases consist of ACN (A) and H_2_O (B) were delivered at a flow rate of 550 μL/min employing isocratic elution mode (A:82% and B:18%). The mass spectrometric detection was carried out in negative ionization applying Selected Reaction Monitoring (SRM) mode including the most abundant and specific Q1 → Q3 ion transitions: 303.1 → 259.3, 311.3 → 267.2 and 314.2 → 270.4 for AA, AAd8 and AAd11 (internal standard), respectively. The sample clean-up protocol was the same as applied for eicosanoid determination.

### Assessment of endothelial function in vivo by MRI

To assess the endothelial dysfunction in the postprandial phase olive oil was administered into C57BL/6 mice and the endothelial function was analyzed 6 h after olive oil administration using unique MRI-based analysis in vivo. The role of vascular ATGL-cPLA2-PGI_2_-dependent pathway activated by lipid overload was assessed by analyzing the functional effects of ATGL-dependent lipolysis in the presence of ATGL inhibitor (atglistatin, Cayman Chemical 200 µmol/kg bw.), cPLA_2_ inhibitor (AACOCF3, 10 mg/kg. Cayman Chemical) and selective PGI_2_ receptor antagonist (RO3244794, 50 mg/kg synthesized by J. Młynarski, Prof, from the Polish Academy of Sciences, Warsaw, Poland) administered 3 h after olive oil gavage.

MRI experiments were performed using a 9.4T scanner (BioSpec 4/20 USR, Bruker, BioSpin GmbH, Germany), as described previously [26]. Mice were anesthetized using isoflurane (Aerrane, Baxter Sp. z o. o., Warszawa, Poland, 1.7 vol. %) in an oxygen and air (1:2) mixture. Body temperature was maintained at 37 °C using circulating warm water. ECG, espiration and body temperature were monitored using a Model 025 Monitoring and Gating System (SA Inc., Stony Brook, NY, United States). Endothelium-dependent vasodilation was assessed in vivo based on the aorta response induced by acetylcholine (Ach) administration, as described previously [27, 28]. Response to injection of Ach (Sigma–Aldrich, Poznan, Poland: 50 μl, 16.6 mg/kg, i.p.), was analyzed in the lower part of the thoracic aorta (ThA) and the abdominal aorta (AbA). Vasomotor response was examined based on time-resolved 3D images of the vessels prior to and 25 min after intraperitoneal Ach administration [26]. Images were acquired using the cine IntraGateTM FLASH 3D sequence, reconstructed with the IntraGate 1.2.b.2 macro (Bruker). End-diastolic volumes of vessels were analyzed using ImageJ software 1.46r (NIH, Bethesda, MD, United States) and scripts written in Matlab (MathWorks, Natick, MA, United States). Imaging parameters included the following: repetition time (TR)—6.4 ms, echo time (TE) – 1.4 ms, field of view (FOV)—30 mm × 30 mm ×  14 mm, matrix size—256 × 256 × 35, flip angle (FA)—30°, and number of accumulations (NA)—15, reconstructed to seven cardiac frames. Total scan time was 10 min.

### Endothelium permeability measurement in vitro

The response of the barrier formed by HAEC to OA (100 µM) in the presence or absence of atglistatin (50 µM) was assessed in real-time in a fully standardized manner by continuously recording changes in electrical resistance with an ECIS system [27], using 96W10E + electrode chamber arrays and an ECIS Z-Theta system (Applied Biophysics), along with the associated software v.1.2.126 PC. Immediately after HAEC seeding, resistance (Ω) measurements were initiated. Once stable resistance was reached, HAEC cells were subjected to treatment with OA alone or in combination with atglistatin for 24 h and the endothelial-barrier resistance was measured in real-time at multiple frequency modes (ranges between 250 and 64,000 Hz). The experiment was performed in a humidified 5% CO_2_ incubator at 37 °C and was repeated 3 times in seven technical replicates [29].

### Statistical analysis

All of the data obtained are presented as mean and standard deviation (SD) or in case of the lack of normal distribution as median with interquartile range. Statistical tests were done using GraphPad Prism 9 (GraphPad Software, Inc., La Jolla, CA, United States) software. Non-parametric test (Kruskal–Wallis test followed by Dunn’s post hoc test) or parametric test (one-way ANOVA followed by Tukey’s or Bonferroni’s post hoc test) were performed. Statistical significance was defined as *p* < 0.05.

## Results

### Exogenous AA induces LDs formation and ATGL- and cPLA_2_-dependent PGI_2_ release

**Fig. 1:**
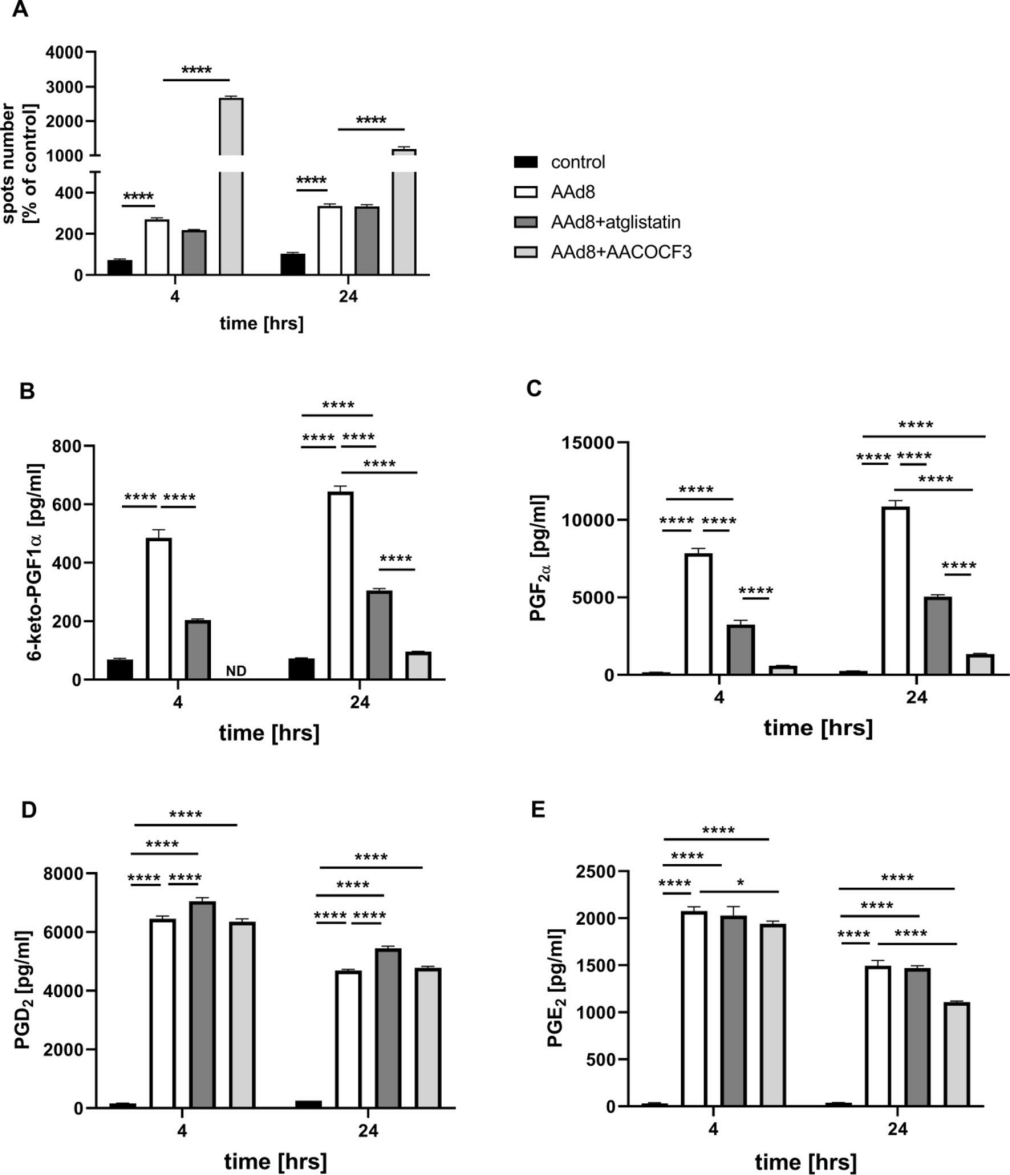
Lipid droplets formation in human aortic endothelial cells (HAEC) induced by exogenous AA (AAd8) and ATGL- and cPLA_2_-dependent eicosanoid release from endogenous AA. Effect of inhibition of atglistatin and AACOCF3 on lipid droplets formation (**A**) and eicosanoids release (**B**–**E**) in HAEC  4 h and 24 h after deuterated arachidonic acid (AAd8, 25 µM) addition in the presence or absence of atglistatin (50 µM) and AACOCF3 (10 µM). Data represent mean ± SD of three independent experiments. Statistical analysis was performed using one-way ANOVA followed by Tukey’s multiple comparisons test (*p < 0.05, ****p < 0.0001). ND - not detected

Incubation of endothelial cells with exogenous AA (AAd8), in non-toxic concentration, (Fig. S3 supplementary materials) resulted in LDs formation that was significant 4 h after AAd8 administration and further slightly increased after 24 h of AAd8 incubation (Fig. 1A). AA-induced LDs formation was associated with elevated production of PGI_2_ as evidenced by increased 6-keto-PGF_1α_ concentration 4 h after AAd8 administration, that further slightly increased after 24 h of AAd8 incubation (Fig. 1B).

LDs formation was also associated with a considerable activation of PGF_2α_ production that increased 4–24 h after AAd8 administration with a similar kinetics as that of 6-keto-PGF_1α_ (Fig. 1C). Interestingly, AA induced also an increase in PGD_2_ and PGE_2_ production (Fig. 1D, E). In the presence of ATGL inhibitor, atglistatin (50 µM), the number of AA-induced LDs in the endothelial cell was not significantly altered, but the concentration of 6-ketoPGF_1α_ (Fig. 1A, B) was significantly lowered. Similarly, AACOCF3, the inhibitor of cytosolic phospholipase A_2_ inhibited the production of PGI_2_ and lowered 6-keto-PGF_1α_ level (Fig. 1B). However, in contrast to the effects of atglistatin, in the presence of AACOCF3, the number of AA-induced LDs was increased (Fig. 1A).

Effects of atglistatin and AACOCF3 in smooth muscle cells were similar as in endothelial cells. AA-induced increase in 6-keto-PGF_1α_ concentration was inhibited. Furthermore, the number of AA-induced LDs was increased 24 h after AAd8 administration (Fig. S1A, B, supplementary materials).

Interestingly, there were divergent effects of atglistatin and AACOCF3 on other eicosanoids released by AA either in endothelial cell (Fig. 1C–E) or in smooth muscle cells (Fig. S1C, D, supplementary materials). While these inhibitors inhibited PGF_2α_ formation to a similar extent as 6-keto-PGF_1α_, their effects on PGD_2_ and PGE_2_ formation, were absent or minimal.

**Fig. 2:**
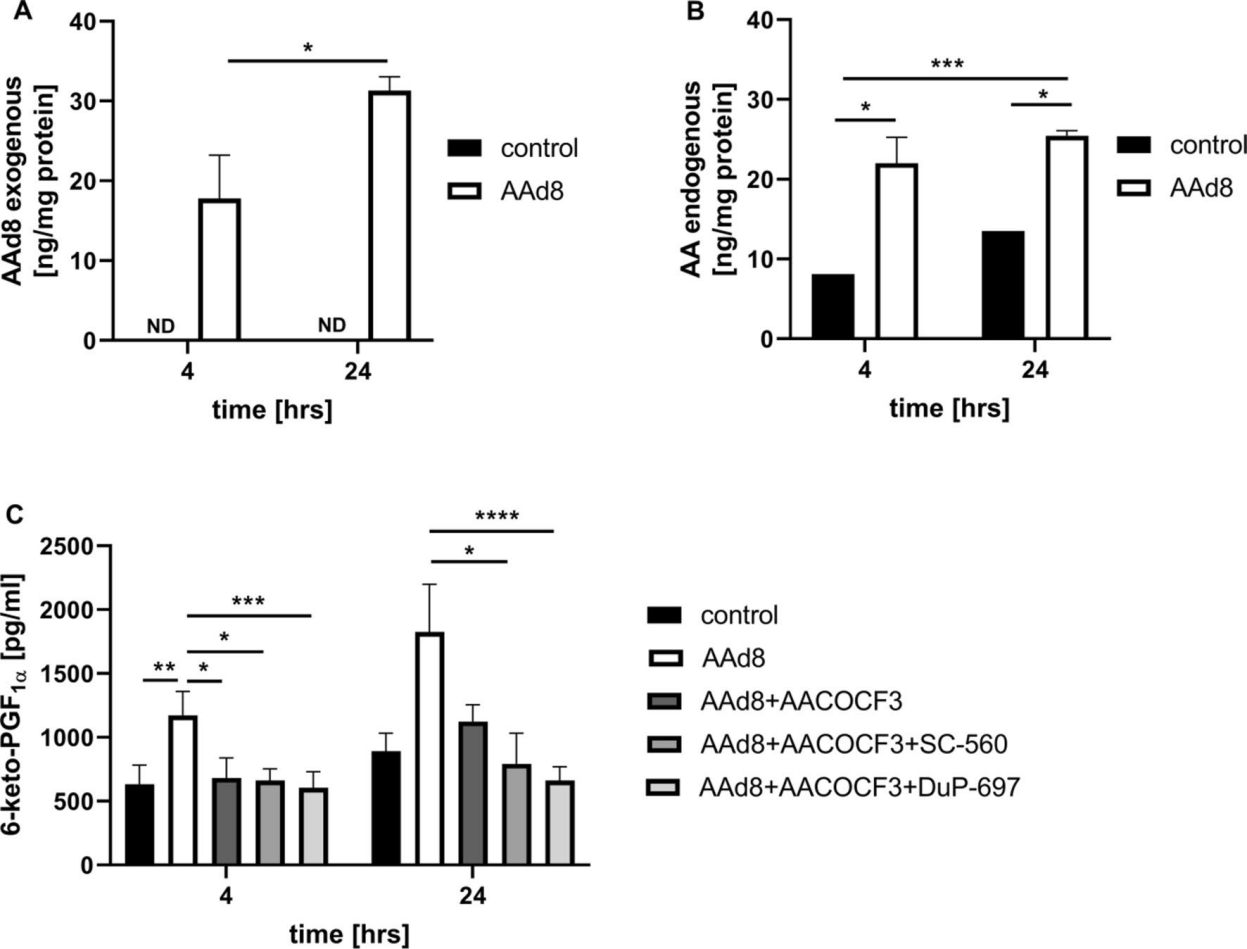
The content of egxogenous AAd8 and endogenous AA in lipid extracts and synthesis of PGI_2_ from endogenous AA by cPLA_2_/COX-1/COX-2 pathway in response to exogenous AAd8 in human aortic endothelial cells (HAEC). The content of exogenous deuterated arachidonic acid (AAd8, 25 µM) (**A**), endogenous AA (**B**) in lipid extracts and the concentration of 6-keto-PGF_1α_ (**C**) in medium in the presence or absence of cPLA_2_, COX-1, COX-2 inhibitors (AACOCF3, SC-560, DuP-697) after 4 h and 24 h of HAEC incubation with AAd8. Data represent mean ± SD of three independent experiments. Statistical analysis was performed using the nonparametric Kruskal–Wallis test followed by Dunn’s multiple comparisons test (*p < 0.05, **p < 0.01, ***p < 0.001, ****p < 0.0001). ND - not detected

 As shown in Fig. 2A and B the increased concentration of both exogenous AAd8 and endogenous AA were observed in lipid extracts after 4 h and 24 h of incubation of HAEC with AAd8. Moreover, AAd8 induced a significant increase in 6-keto-PGF_1α_ concentration that was inhibited by AACOCF3, SC-560 and DuP-697 (inhibitors specific for cPLA_2_, COX-1 and COX-2, respectively) suggesting that exogenous AA induced PGI_2_ synthesis from endogenous AA that was mediated by cPLA_2_/COX pathway involving COX-1 and COX-2 (Fig. 2C).

### Exogenous OA induces LDs formation and ATGL- and cPLA_2_-dependent PGI_2_ release

Similarly, to exogenous AA, deuterated oleic acid (OAd34), in the non-toxic concentration, (Figs. S2, S3 supplementary materials) induced LDs formation and PGI_2_ release in endothelial cells and vascular smooth muscle cells.

**Fig. 3:**
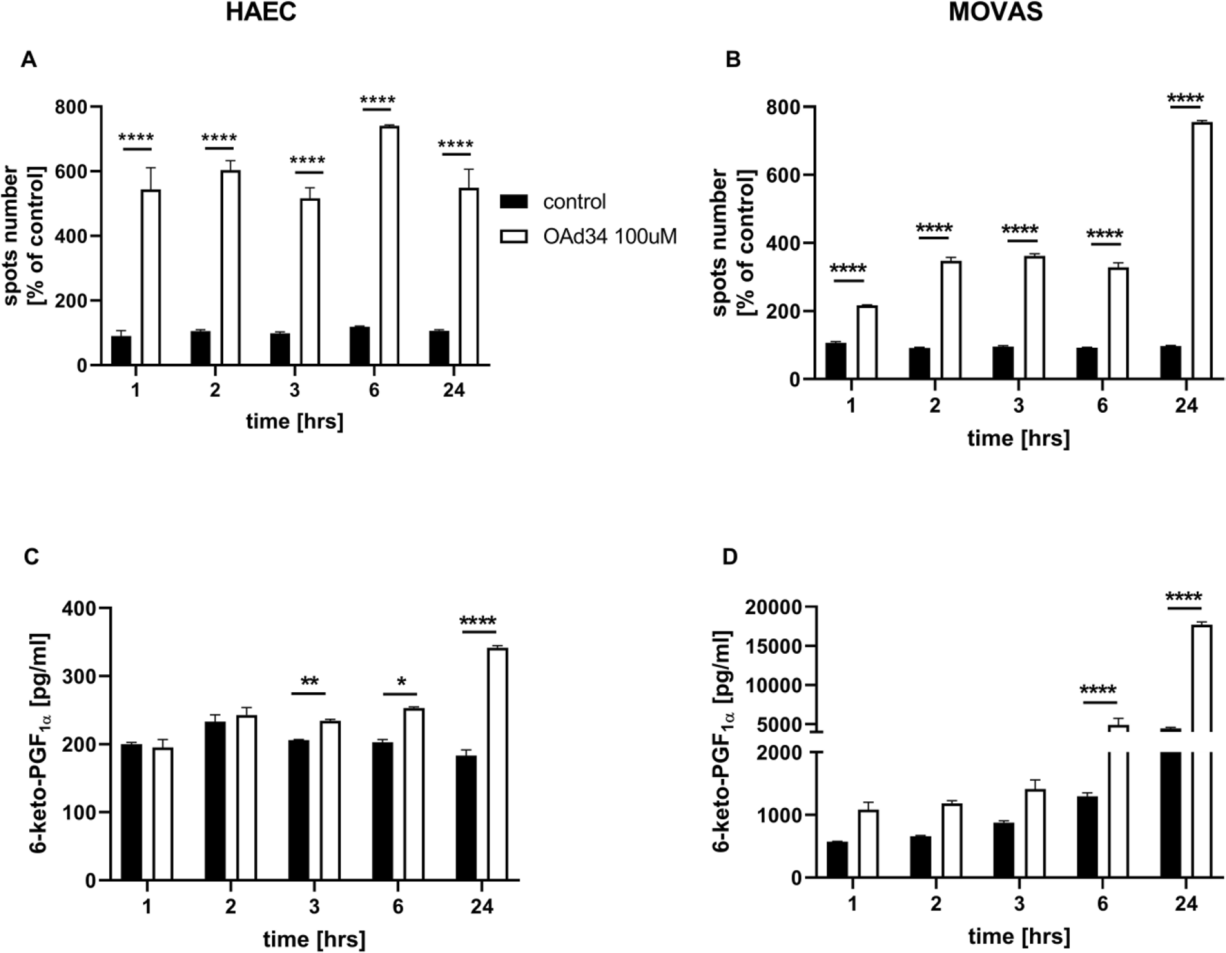
Lipid droplets formation in human aortic endothelial cells (HAEC) and vascular smooth muscle cells (MOVAS) induced by exogenous OA; parallel formation of PGI_2_ from endogenous AA in vitro.Time course of changes in lipid droplets formation (**A**, **B**) and 6-keto-PGF_1α_ release (**C**, **D**) stimulated by deuterated OA (OAd34, 100M, 1 h, 2 h, 3 h, 6 h, 24 h). Data represent mean ± SD of three independent experiments. Statistical analysis was performed using one-way ANOVA followed by Tukey’s or Bonferroni’s multiple comparisons test (*p < 0.05, **p < 0.01, ****p < 0.0001)

As shown in Fig. 3, incubation of endothelial cells with deuterated oleic acid (OAd34) increased the number of LDs. The effect was already seen 1 h after OAd34 addition and remained at similar level 2, 3, 6, and 24 h after OAd34 administration to endothelial cells (Fig. 3A).

In smooth muscle cells number of LDs induced by OAd34 was significantly higher after 24 h as compared to shorter incubation times (Fig. 3B).

Parallel to the increase in LDs formation, the 6-keto-PGF_1α_ concentration was elevated in either endothelial cells or in smooth muscle cells. However, a significant increase in 6-keto-PGF_1α_ concentration induced by OAd34 in HAEC or MOVAS, was delayed as compared with rapid LDs formation and was most substantially increased 24 h after OAd34 administration (Fig. 3C, D). Interestingly, the concentration of 6-keto-PGF_1α_ released by OAd34 was higher in MOVAS as compared with HAEC (Fig. 3D).

To further confirm that exogenous OA resulted in LDs formation that was associated with PGI_2_ release biochemical composition of LDs induced by OA and 6-keto-PGF_1α_ released was studied in isolated aorta en face.

**Fig. 4:**
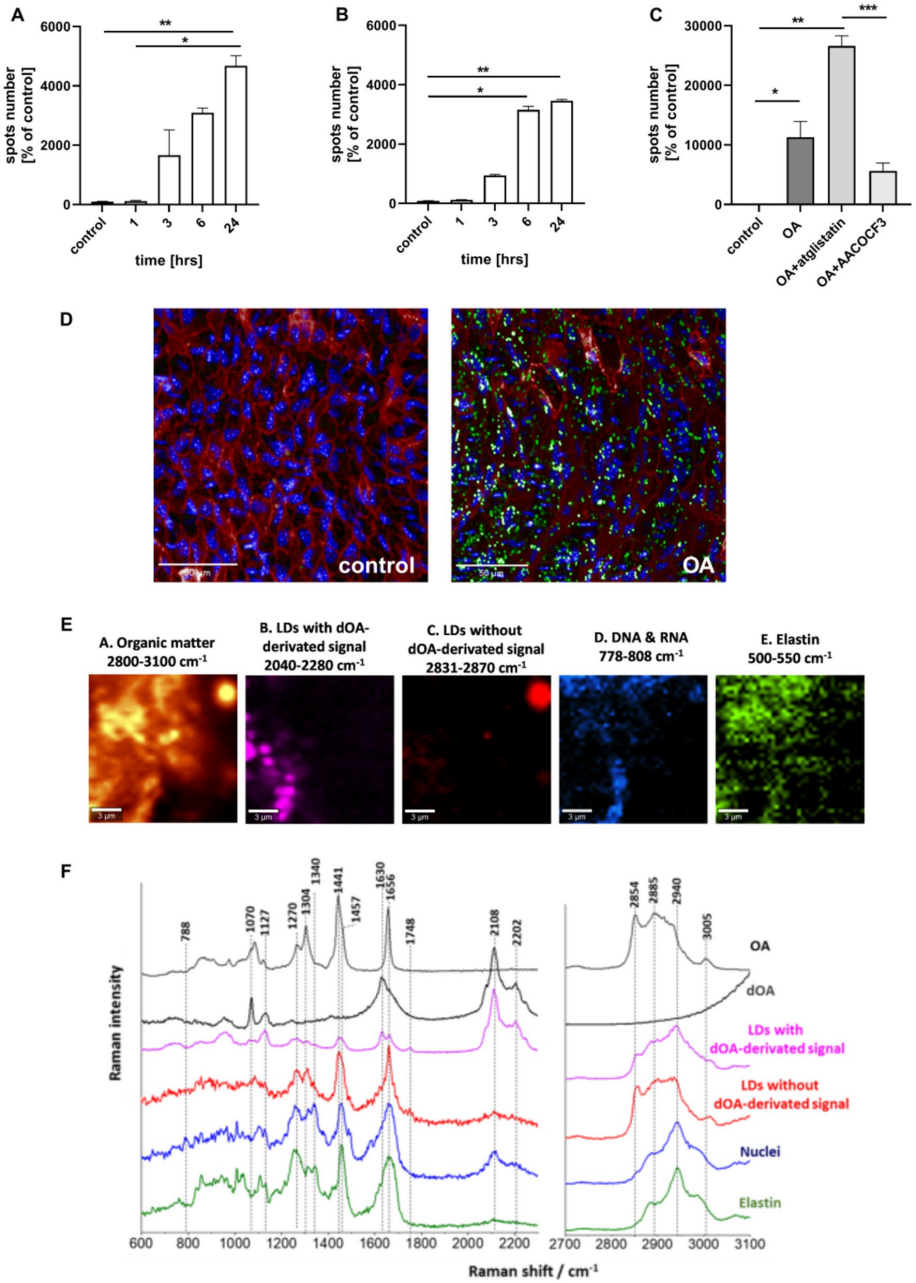
Lipid droplets formation in isolated murine aorta in response to exogenous OA. Time course of changes in lipid droplets formation in endothelium (**A**) and smooth muscle cells (**B**) within isolated murine aorta stimulated by deuterated oleic acid (OAd34, 1 mM; 1 h, 2 h, 3 h, 6 h, 24 h). The data are presented as the median with interquartile range (*n* = 4). Statistics: Kruskal–Wallis test followed by Dunn’s multiple comparisons test. (*p< 0.05, **p < 0.01 ). Effect of inhibition of atglistatin and AACOCF3 on lipid droplets formation in isolated murine aorta ex vivo stimulated by OA (500 µM, 24 h) in the presence or absence of atglistatin (50 µM) and AACOCF3 (10 µM)(**C**). The data are presented as the median with interquartile range (*n* = 6–8). Statistics: Kruskal–Wallis test followed by Dunn’s multiple comparisons test (*p < 0.05, **p < 0.01, ***p < 0.001). Representative microphotographs of immunostaining of control en face aorta and aorta treated with OA (1 mM, 24 h). Red, blue and green fluorescence originating from PECAM-1, cell nuclei and LDs, respectively (**D**). Visualisation of biochemical components of murine en face aorta with LDs formed in response to OAd34 (1 mM, 24 h) by Raman spectroscopy (brighter colours characterizing higher intensities of respective bands). Apart from typical structures like nuclei or elastin fibres Raman imaging of en face aorta show two kinds of lipid droplets: rich in OAd34 and without OAd34 (**E**, ** F**) but most likely containing AA

As shown in Fig. 4, OA induced LDs formation in the endothelial layer as well as in smooth muscle cells in the isolated aorta as evidenced by fluorescence staining. The number of LDs in endothelium was significantly higher 24 h after OA administration (Fig. 4A).

Interestingly, in smooth muscle the increased LDs number was observed already 6 h after OAd34 administration and remained at a high level also 24 h after OAd34 administration (Fig. 4B).

In the presence of atglistatin, but not AACOCF3, the OA-induced LDs formation in the aorta was significantly increased (Fig. 4C). Raman spectroscopy confirmed the presence of LDs in endothelium and in deeper layers of the vessel wall after incubation with OAd34, and identified LDs rich in exogenous oleic acid (OAd34) uptaken from medium and LDs containing more unsaturated lipids then OAd34, most likely AA (Fig. 4E, F).

**Fig. 5:**
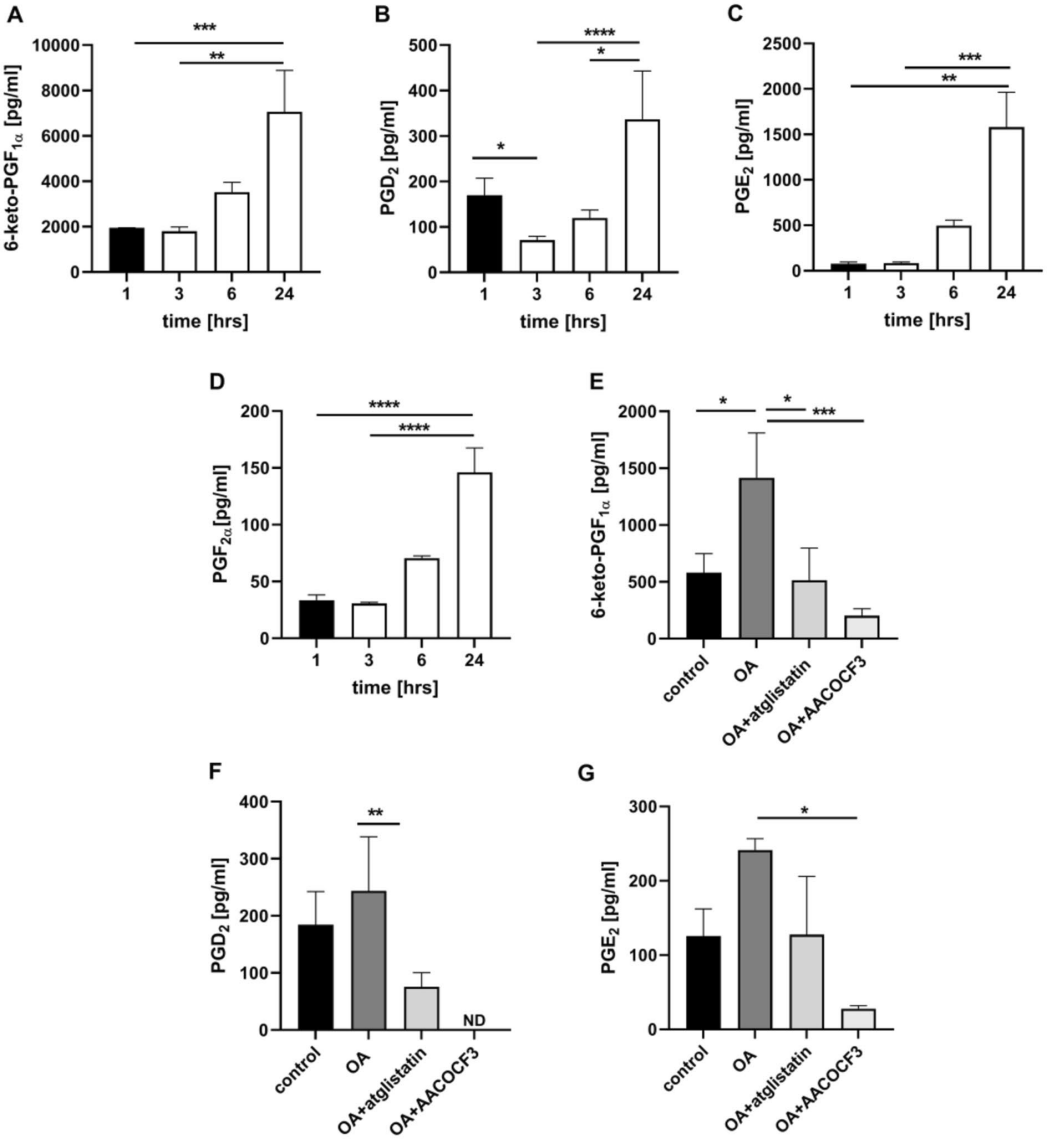
ATGL- and cPLA_2_-dependent PGI_2_ release from endogenous AA in isolated murine aorta in response to exogenous OA. Time course of changes in eicosanoids release in endothelium within isolated murine aorta stimulated by deuterated oleic acid (OAd34, 1 mM; 1 h, 2 h, 3 h, 6 h, 24 h) (**A**–**D**). The data are presented as the median with interquartile range (*n* = 6). Statistics: Kruskal–Wallis test followed by Dunn’s multiple comparisons test (*p< 0.05, **p < 0.01, ***p < 0.001, ****p < 0.0001). Effect of inhibition of atglistatin (50µM) and AACOCF3 (10 µM) on eicosanoids release in isolated murine aorta ex vivo stimulated by OA (500 µM, 24 h) (**E**–**G**). The results are presented as the mean ± SD (*n* = 6–14). Statistics: one-way ANOVA followed by Tukey’s or Bonferroni’s multiple comparisons test) (*p < 0.05, **p < 0.01, ***p < 0.001, ****p < 0.0001)

Similarly to the experimental system of isolated endothelial cells (Fig. 3A, C) and smooth muscle cells (Fig. 3B, D), OA-induced LDs in isolated murine aorta was associated with a time-dependent PGI_2_ production as evidenced by 6-keto-PGF_1α_ release that was most pronounced 24 h after OAd34 administration (Fig. 5A). Again, atglistatin and AACOCF3 inhibited OA-induced PGI_2_ production as well as other prostanoids release (6-keto-PGF_1α_, PGD_2_, PGE_2_) indicating that ATGL and cPLA_2_ are required for LDs metabolism in vascular wall and mediate the production of vascular PGI_2_ in response to exogenous OA (Fig. 5E–G).

### ATGL-dependent lipolysis regulates endothelial barrier function

**Fig. 6:**
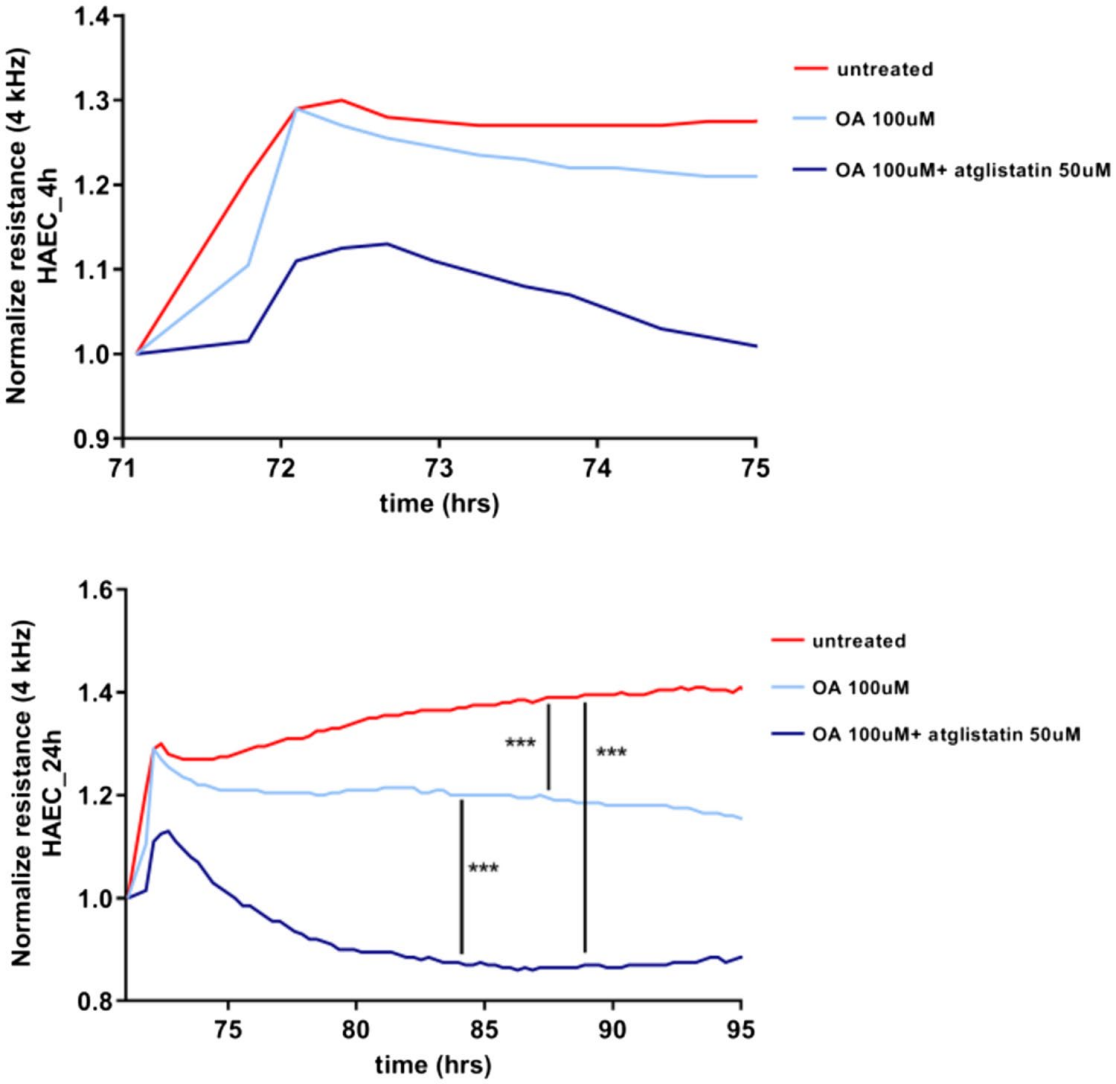
Effects of the inhibition of ATGL-dependent-lipolysis of LDs induced by exogenous OA on the regulation of the endothelial barrier integrity in vitro. Effect of inhibition of atglistatin (50 µM) on permeabililty changes in HAEC 4 h and 24 h after OA administration (100 µM) measured by using ECIS method. Data represent mean ± SD of three independent experiments Statistics: one-way ANOVA followed by Tukey’s multiple comparisons test (***p < 0.001)

The functional role of ATGL-dependent lipolysis induced by OA was analyzed by studying the effects of atglistatin (50 µM) on OA (100 μM)-induced changes in endothelial barrier permeability in an in vitro assay. As shown in Fig. 6, OA alone did not induce significant variation in the cells’ monolayer resistance as assessed by ECIS, however, the combination of OA with atglistatin caused a decrease in the resistance of the endothelial cells, indicating impairment of the endothelial barrier integrity (Fig. 6).

### ATGL-dependent lipolysis and the activation of cPLA_2_-PGI_2_-dependent pathway protects endothelial function in postprandial phase

**Fig. 7:**
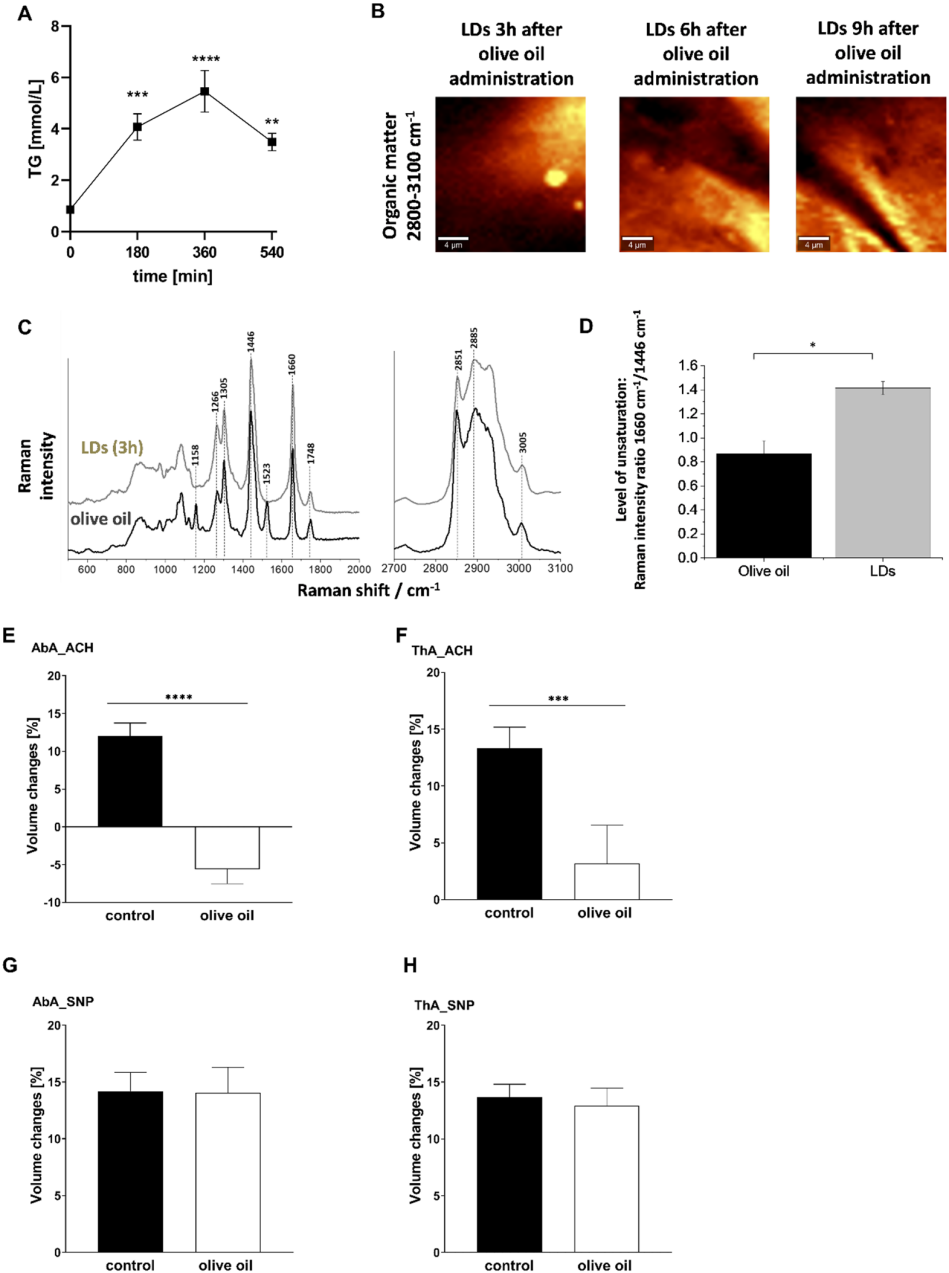
Postprandial formation of LD and development of endothelial dysfunction in vivo in aorta associated with increased TG plasma level induced by olive oil gavage. C57BL/6 mice were fasted for 16 h before olive oil administration. The dose of olive oil was chosen based on plasma TG measurement with plasma TG levels peak at 360 min (**A**). The results are presented as the mean ± SD (*n* = 4–6). Statistics: one-way ANOVA followed by Tukey’s multiple comparisons test (**p < 0.01, ***p < 0.001, ****p < 0.0001). Raman imaging of lipid droplets formation in murine aorta isolated from mice 3 h, 6 h and 9 h after intra-gastric administration of the olive oil (10 mL/mg) (**B**). Comparison of the spectrum of the olive oil with the average spectrum of endothelial LDs of an isolated blood vessel 3 h, after intra-gastric administration of the olive oil. The Raman spectrum of LDs indicates higher lipid unsaturation than the administered olive oil as the ratio of the bands 1660 to 1446 cm^−1^, and 1266 to 1305 cm^−1^ is higher for the LDs spectrum than for olive oil (**C, D**). Endothelial function after olive oil administration—assessed in vivo using unique MRI-based analysis (**E**). Changes in end-diastolic volume of the abdominal (**A**:AbA) and thoracic aorta (**B**: ThA) 30 min after acetylcholine administration or 30 min after sodium nitroprusside administration (**C**: AbA;** D**: ThA) in C57BL/6 mice gavaged with olive oil (10 mL/kg). Measurements were placed 6 h after the olive oil gavave. The dose of olive oil was chosen based on the plasma triglycerides measurement curve. The results are presented as the mean ± SD (*n* = 6–14). Statistics: one-way ANOVA followed by Tukey’s multiple comparisons test (*p < 0.05, ***p < 0.001)

The possible vasoprotective role of vascular ATGL-cPLA_2_-PGI_2_-dependent pathway activated by lipid overload was confirmed in in vivo model of postprandial endothelial dysfunction induced by olive oil (10 mL/kg) in vivo. The dose of olive oil was chosen based on plasma triglycerides measurements with a clear-cut peak of plasma TG levels 360 min after olive oil administration (Fig. 7A). Vascular LDs detected via fluorescence and Raman imaging were observed 180 min after olive oil administration but were not visible at later time points (Fig. 7B, C, D). Raman spectrum of LDs indicated a higher lipid unsaturation than the administered olive oil as the ratio of the bands 1660 to 1446 cm^−1^, and 1266 to 1305 cm^−1^ was higher for the LDs spectrum than for olive oil. Interestingly, the fatty acid profile measured in isolated aorta wall by chromatography-mass spectrometry displayed changes after olive oil administration in unsaturated fatty acids: oleic (18;1) and palmitoleic acid (16:1) as well as linoleic acid (18:2, converted from oleic acid) were higher after 9 h as compared to 6 h after olive oil suggesting lipolysis (data not shown).

**Fig. 8:**
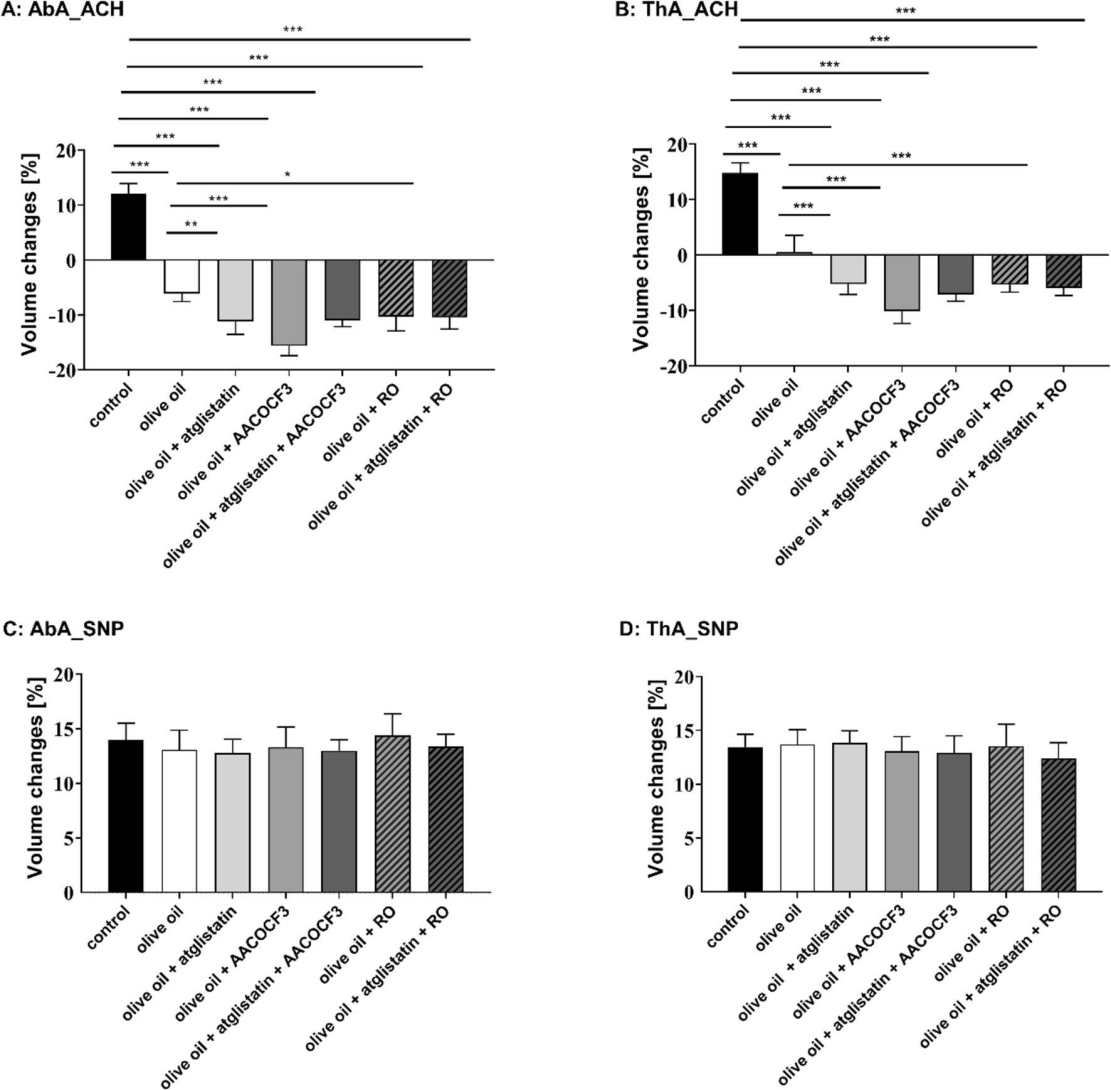
Protective role of vascular ATGL-dependent lipolysis and the activation of cPLA_2_–PGI_2_ pathway in the postprandial endothelial dysfunction induced by olive oil gavage. Atglistatin (200 µg/mL), AACOCF3 (10 mg/kg) and RO 3244794 (50 mg/kg) were used to inhibit ATGL, cPLA_2_ or to block IP receptor respectively. Endothelial function after olive oil administration was assessed in vivo using unique MRI-based analysis (**A**–**C**). Changes in end-diastolic volume of the abdominal (**A**: AbA) and thoracic aorta (**B**: ThA) were measured 30 min after acetylcholine administration or 30 min after sodium nitroprusside administration (**C**: AbA; **D**: ThA) in C57BL/6 mice gavaged with olive oil (10 mL/kg). Measurements were performed 6 h after olive oil gavage. Statistics: one-way ANOVA followed by Tukey’s multiple comparisons test (*p < 0.05, **p < 0.01, ***p < 0.001)

On the functional level, as shown in Fig. 7E–H, olive oil administration induced endothelial dysfunction as evidenced by impaired Ach-induced endothelium-dependent vasodilation in the thoracic and in the abdominal aorta in C57BL/6 mice 6 h after olive oil administration. In the presence of each inhibitor administered alone, atglistatin (200 µmol/kg), administered together with olive oil, AACOCF3 (cPLA_2_ inhibitor, 10 mg/kg) or RO3244794 (selective PGI_2_ receptor antagonist, 50 mg/kg) administered 3 h after olive oil gavage, Ach-induced response was further deteriorated (Fig. 8A, B) suggesting that postprandial endothelial dysfunction was aggravated by ATGL, cPLA_2_ or IP receptor blockade in vivo. Interestingly, combined blockade (atglistatin with AACOCF3 or RO3244794) did not result in further deterioration of the Ach-induced response as compared with a single inhibitor given alone, suggesting that each inhibitor blocked the same pathway. In contrast, sodium nitroprusside (SNP)-induced endothelium-independent response in the thoracic and abdominal aorta was not affected by olive oil gavage (Fig. 7G, H) and by atglistatin, AACOCF3 or RO3244794 (Fig. 8C, D) given alone or in combination, which confirms specific protective effects of ATGL-dependent lipolysis and the activation of cPLA_2_-PGI_2_ pathway on endothelial function in postprandial phase after olive oil administration.

## Discussion

In the present work, we demonstrated that ATGL-dependent lipolysis maintains vascular homeostasis by cPLA_2_-dependent PGI_2_ production from endogenous AA. Our results indicated that lipid overload of the vascular wall or endothelial/smooth muscle cells alone resulted in LDs formation that was tightly linked with the activation of endogenous ATGL-cPLA_2_-AA-PGI_2_ pathway to protect against detrimental effects of lipid overload on endothelial function. In particular, we demonstrated that vascular ATGL-dependent lipolysis and the activation of cPLA_2_–PGI_2_ pathway protects endothelial function against lipids overload in the postprandial phase. Accordingly, the inhibition of ATGL suggested to be a therapeutic target in liver inflammation and NAFLD [2], myocardial injury [4, 5] or heart failure [6, 7] may lead to detrimental effects on endothelial function. Similarly, impaired vascular ATGL activity induced by constitutive or genetic factors [30] may contribute to the deterioration in endothelial function and its clinical consequences.

It is well known that endothelium and smooth muscle cells are able to produce PGI_2_ and vascular PGI_2_ has vasoprotective, anti-thrombotic or anti-proliferative effects linked to cPLA_2_-COX-1-COX-2 pathway. However, little was known on the relationship between ATGL-dependent lipolysis and the activation of cPLA_2_-PGI_2_ pathways. Although previous reports demonstrated that LDs formation in endothelium was linked to PGI_2_ generation [15, 31] the functional consequences on endothelial function and pathways involved were not elucidated. Here, we clearly demonstrated that exogenous OA or AA-induced the formation of LDs and the LDs formation in endothelial cells, smooth cells or in the isolated aorta was associated with subsequent hydrolysis of AA-rich triglycerides stored in LDs and elevated production of endogenous AA-derived PGI_2_ and other eicosanoids. Furthermore, in our hands, the inhibition of cPLA_2_ or in some experimental settings, the inhibition of ATGL was associated with the increased number of LDs, suggesting that the formation of vascular LDs is dynamically linked with ATGL-cPLA_2_-dependent lipolysis and the generation of eicosanoids. The major product of ATGL-cPLA_2_-dependent lipolysis was PGI_2_ but other eicosanoids were also generated. Interestingly, while PGI_2_ was inhibited by atglistatin in endothelial cells and smooth muscle cells, this effect was not observed in relation to PGD_2_ and PGE_2_ suggesting that LDs formation in endothelium or smooth muscle cells is linked to the major vascular metabolite of arachidonic acid pathway PGI_2_ rather than to PGD_2_ and PGE_2_ known to be regulated by distinct mechanisms as compared with PGI_2_ [32, 33]_._

In recent studies the relationship between increased PGI_2_ production and LDs formation was reported for the high concentration of exogenous free fatty acids: oleic [12] or arachidonic [34]. Increased generation of PGI_2_ concomitant to LDs formation in the vascular wall in response to OA and other pro-inflammatory factors was also reported recently by our group [24]. In the present work, however, we demonstrated that LDs formation was linked in the time-dependent manner with subsequent ATGL-cPLA_2_-dependent lipolysis and the release of PGI_2_ that was generated from endogenous AA. This scenario pertained to endothelial cells, smooth cells in culture, intact isolated aorta and was shown to have a functional role in vivo during a postprandial phase.

In the present work, we used a couple of experimental approaches to demonstrate that the release of PGI_2_ originated from endogenous sources of AA, independently of whether AA or OA was used as a stimulus for LDs formation. First, to confirm whether eicosanoids were generated from endogenous AA, exogenous deuterated AA or deuterated OA were used. Using this approach, we demonstrated that deuterated AA induced a significant increase in 6-keto-PGF_1α_ concentration comparable to the effect induced by non-deuterated AA. Moreover, we demonstrated that 6-keto-PGF_1α_ release by deuterated AA was inhibited by AACOCF3, SC-560 and DuP-679 (inhibitors specific for cPLA_2_, COX-1 and COX-2, respectively) suggesting that exogenous AA-induced PGI_2_ synthesis from endogenous AA that was mediated by cPLA_2_/COXes pathway.

Furthermore, similarly, to exogenous AA, also exogenous OA induced LDs formation and cPLA_2_-dependent PGI_2_ release generated from endogenous AA in endothelial cells, vascular smooth muscle cells as well as isolated vessel. Importantly, Raman imaging used in our study further pointed out the possible presence of endogenous AA in LDs in isolated aorta formed in the response to deuterated OA. In fact, LDs contained unsaturated lipids that could be attributed to AA, but definitely not to deutereated OA [35].

The biochemical content of endothelial LDs was previously, studied by our group in the cultured endothelial cells (HAEC) and in the isolated aorta, upon incubation with deuterated OA. As the major component, OA was revealed (80–90%) with AA contribution (8.7–19.4%) [13]. Altogether, based on these results we claim that the formation of LDs even in response to OA and despite an excess of OA was related to the endogenous AA-induced eicosanoids biosynthesis in the vascular wall.

Of note, on the basis of functional assays in vitro and ex vivo we demonstrated the vasoprotective role of ATGL against triacylglycerol overload. As AA stored in the form of triacylglycerols pool can be re-released by the cell [18, 36], the inhibition of ATGL leading to the blockade of LDs decomposition and therefore blocking the AA release from LDs reduces the availability of AA to eicosanoid production including PGI_2_. Indeed, our results showed an increase in the number of LDs along the inhibition of eicosanoids production in response to ATGL inhibitor after exogenous OA or AA treatment. These results highlight ATGL as the new player in PGI_2_ generation related to LDs formation in the vascular wall in response to lipid overload. Moreover, the importance of ATGL for vasoprotection may also be linked to anti-platelet, anti-inflammatory and anti-atherosclerotic activity of PGI_2_ [37, 38].

The important aspect of this work was to demonstrate the vasoprotective activity of ATGL-dependent lipolysis and subsequent activation of cPLA_2_-PGI_2_ pathway in the context of postprandial endothelial dysfunction induced by olive oil administration, a well-known model of postprandial response. The novelty of our approach was to take advantage of our unique approach using MRI [26] to measure endothelial function in vivo in the postprandial phase. We set the animal model of postprandial endothelial dysfunction to show that parallel to maximal serum TG concentration the endothelial dysfunction developed as evidenced by impaired Ach-induced vasodilation in the thoracic and abdominal aorta, with concomitant presence of LDs in the early but not late phase of postprandial response confirming the dynamics of LDs formation and lipolysis in postprandial phase reported previously [39]. Importantly, our results to the best of our knowledge, for the first time, showed that inhibition of ATGL, cPLA_2_ or blockade of IP receptor in each of the intervention alone resulted in deterioration of endothelial function upon exposure to lipids during the postprandial phase. Of note, combined blockade of any two of the target (atglistatin with AACOCF3 or RO3244794) did not result in the further deterioriation of postrandial endothelial dysfunction as compared with the single inhibitor. These results pointed out that ATGL-dependent lipolysis, and subsequent activation of cPLA_2_-PGI_2_ pathway operated together to afford vasoprotective action offsetting detrimental vascular effects of lipid overload during postprandial hyperlipidemia.

In addition, as one of the phenotypic feature of endothelial dysfunction is linked to changes in endothelial permeability [26], we also studied the effects of ATGL-dependent lipolysis on endothelial barrier function in vitro. Similarly to results from in vivo assay, OA-induced endothelial effects in vitro assessed by the electrical cell impedance sensor (ECIS) in terms of endothelial barrier integrity was deteriorated if OA was given together with atglistatin.

It is known since many years that elevated non-fasting triglyceride levels were associated with an increased risk of cardiovascular events [40]. Furthermore, there is an association between postprandial dysmetabolism and both coronary artery disease and cardiac events [41, 42]. Accumulation of triglyceride-rich lipoproteins promotes the development of atherosclerosis [43] and a delayed clearance of postprandial lipoproteins from the plasma was suggested to play a role in the etiology of premature coronary atherosclerosis [44, 45]. Given that humans spend the majority of their day in a postprandial (fed) state, with a continual fluctuation in the degree of lipemia throughout the day [46] the effects of postprandial state on endothelial function may play an important determining role in the development of atherosclerosis and cardiometabolic diseases. A large number of studies have shown that endothelial function assessed as flow-mediated vasodilation (FMD) was affected by postprandial lipemia with various mechanisms proposed including oxidative stress, inflammatory burden and others [47]. Recently, it has been suggested that the formation of LDs protects endothelial cell from lipotoxic stress, regulates EC glycolysis and provides a source of FA for adjacent cells in the vessel wall [12]. Here, we extended these findings and provided direct evidence that vascular ATGL-dependent lipolysis and subsequent activation of cPLA_2_-PGI_2_ preserve endothelial function in the postprandial phase thus, pointing out to the ATGL-cPLA_2_-PGI_2_ pathway to offset excess of fat in the postprandial phase and to afford vasoprotection.

Previous studies have highlighted the role of ATGL in lipid metabolism in regulating various aspects of fatty acid uptake and LDs size [48]. Systemic knockout of ATGL resulted in a distinct murine phenotype that was characterized by cardiac steatosis and severe heart failure as well as impaired endothelial NO synthase expression and activity [49]. LDs formation was shown to protect endothelial cells from lipotoxic stress, providing a source of fatty acids for adjacent cells in the vessel wall or tissues [12]. Finally, it was also shown that in vascular endothelial cells, the efficiency of stimulus-induced AA release and prostacyclin secretion was dependent on ATGL [36]. However, the role of ATGL-induced lipolysis in the regulation of endothelial function during postprandial hyperlipidemia and the mechanistic insight linking LDs formation with the activation of endogenous ATGL-cPLA_2_-AA-PGI_2_ pathway evidenced in the present work shed a novel light into the pathophysiological relevance of the formation and the vasoprotective role of vascular LDs.

In conclusion, in the present work using comprehensive methods including functional assays in vivo and in vitro and mechanistical studies we demonstrated that endogenous ATGL-cPLA_2_-PGI_2_ pathway activated by exogenous lipid overload, associated with LDs formation in endothelium and smooth muscle cells has a vasoprotective role as it counterbalances detrimental vascular effects of lipid overload and thus may play an important role in maintaining endothelial function in the postprandial state. Given the key role of endothelial dysfunction occurring in the postprandial phase in determining the development of atherosclerosis and cardiometabolic diseases, our results have important pathophysiological and pharmacotherapeutic significance that warrant further studies.

### Supplementary Information

Below is the link to the electronic supplementary material.

Supplementary file1 (DOCX 345 KB)

## Data Availability

Not applicable.

## Abbrevation

LDs: Lipid droplets
ATGL: Adipose triglyceride lipase
OA: Oleic acid
AA: Arachidonic acid
AACOCF3: Arachidonyl trifuoromethyl ketone
SC-560: 5-(4-Chlorophenyl)-1-(4-methoxyphenyl)-3-trifluoromethyl pyrazolecyclooxygenase
DuP-697: 5-Bromo-2-(4-fluorophenyl)-3-(4-(methylsulfonyl)phenyl)-thiophene
cPLA_2_: Cytosolic phospholipase A_2_
PGI_2_: Prostacyclin
6-keto-PGF_1α_: 6-Keto-prostaglandin F_1α_
PGD_2_: Prostaglandin D_2_
PGE_2_: Prostaglandin E_2_
PGF_2α_: Prostaglandin F_2α_
