# Supplementary material for: Vascular ATGL-dependent lipolysis and the activation of cPLA2–PGI2 pathway protect against postprandial endothelial dysfunction
Source: Cell Mol Life Sci. 2024 Mar 12;81(1):125. doi: 10.1007/s00018-024-05167-6 (PMC10927860; doi:10.1007/s00018-024-05167-6)
Supplement: Supplementary file 1 — Supplementary file1 (DOCX 345 KB) [file 18_2024_5167_MOESM1_ESM.docx]

**Supplementary materials**


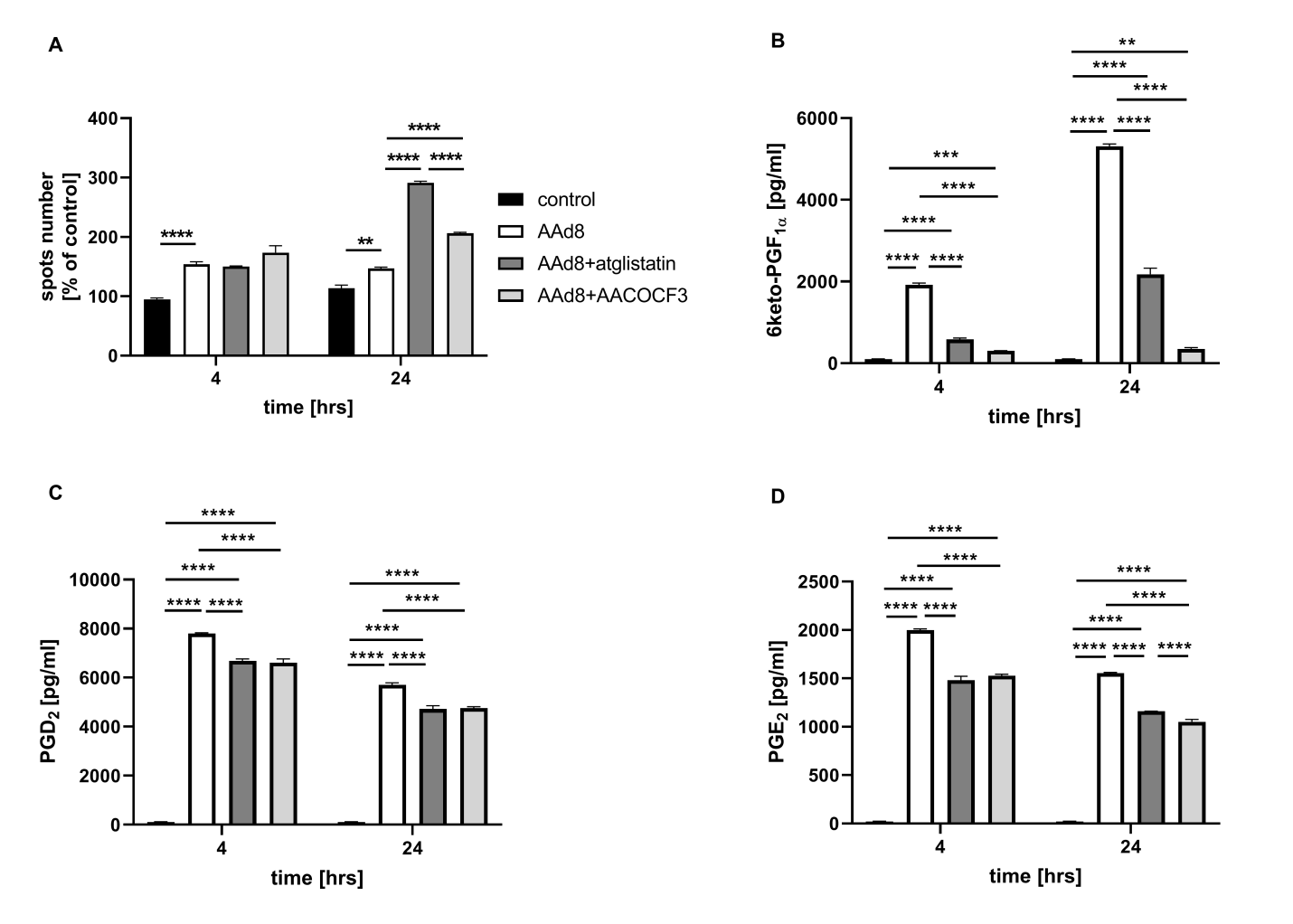


**Fig S1 Lipid droplets formation in smooth muscle cells (MOVAS) induced by exogenous AA (AAd8) and ATGL- and cPLA_2_-dependent eicosanoid release from endogenous AA.**

Effect of inhibition of atglistatin and AACOCF3 on lipid droplets formation and eicosanoids release in MOVAS 4 h and 24 h after deuterated arachidonic acid (AAd8, 25 µM) addition in the presence or absence of atglistatin (50 µM) and AACOCF3 (10 µM). Data represent mean ± SD of three independent experiments. Statistical analysis was performed using one-way ANOVA followed by Tukey’s multiple comparisons test (** p < 0.01, **** p < 0.0001).


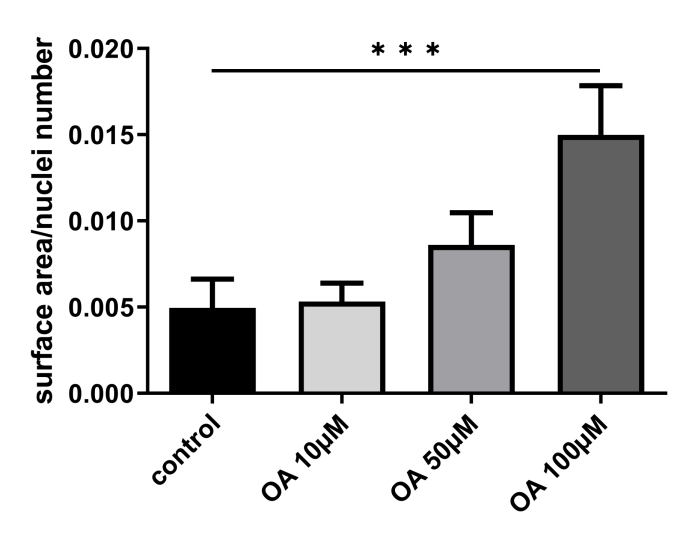


**Fig. S2** **Lipid droplets formation in human aortic endothelial cells (HAEC) induced by exogenous OA at various concentrations (10µM, 50µM, 100µM).**

Data represent mean ± SD of three independent experiments. Statistical analysis was performed using one-way ANOVA followed by Tukey’s multiple comparisons test (*** p < 0.001).


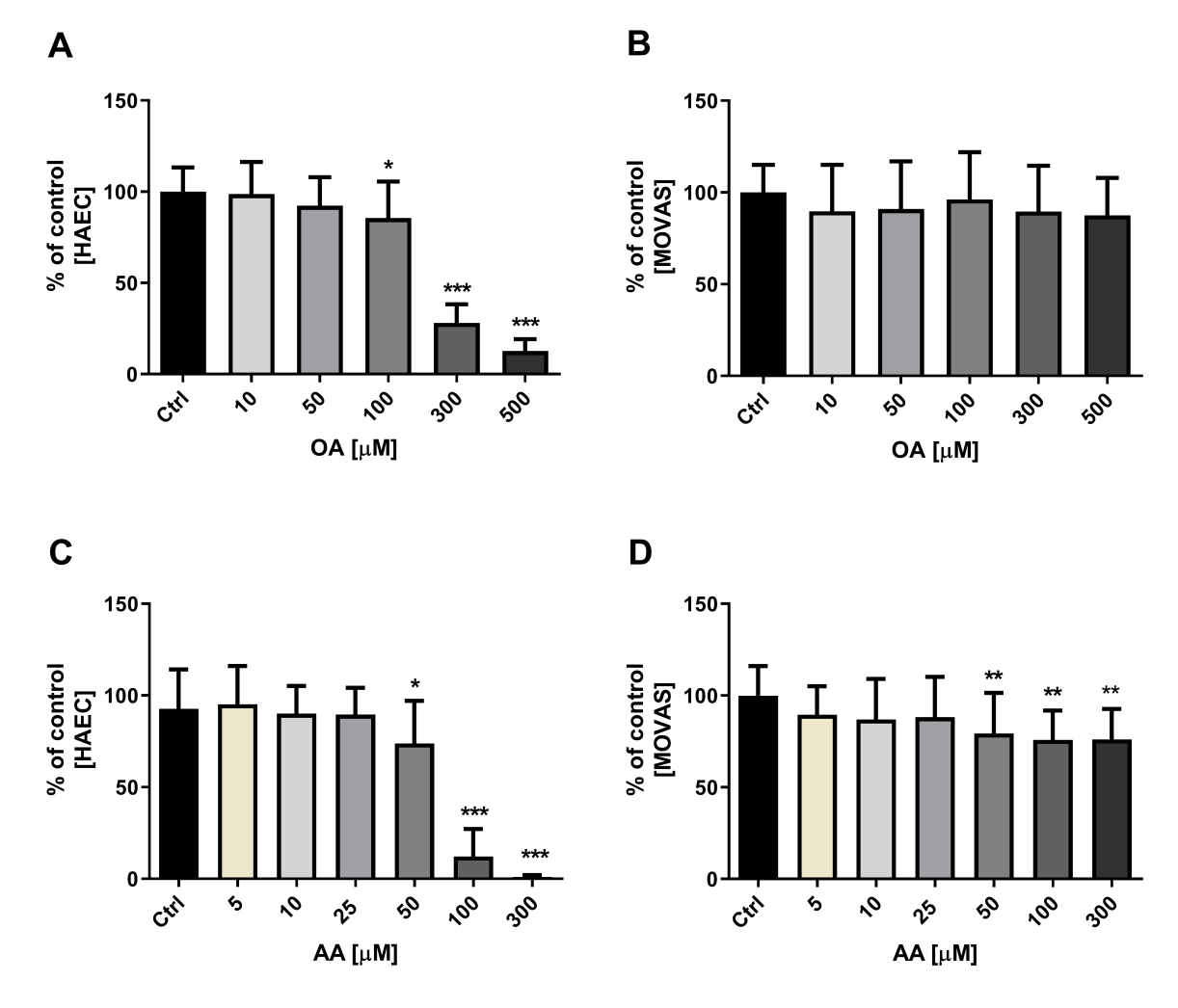


**Fig.S3** **The effect of oleic acid (OA) and arachidonic acid (AA) on HAEC and MOVAS cell viability assessed after 24 hours**. In vitro MTS cytotoxicity assay showing the HAEC (A, C), MOVAS (B, D) cell viability when exposed to OA or AA after 24 h of incubation. Data represent the means ± SD of at least three independent experiments. Statistical analysis was calculated using parametric one-way ANOVA followed by Dunnett's multiple comparisons test (*p < 0.05, **p < 0.01, ***p < 0.001).
